# Airway clearance therapy in acute paediatric respiratory illness: A state-of-the-art review

**DOI:** 10.4102/sajp.v75i1.1295

**Published:** 2019-06-25

**Authors:** Brenda M. Morrow

**Affiliations:** 1 Department of Paediatrics and Child Health, University of Cape Town, Cape Town, South Africa

**Keywords:** chest physiotherapy, physical therapy, paediatric, airway clearance therapy, lower respiratory tract infection, acute

## Abstract

**Background:**

Despite unclear evidence of effectiveness or safety, airway clearance therapy (ACT) is frequently performed in infants and children with acute pulmonary disease.

**Objectives:**

The aim of this review was to critically synthesise published evidence, expert opinion and pathophysiological principles to describe the indications, effects, precautions and application of commonly used ACT modalities for managing infants and children with acute pulmonary disease.

**Method:**

A comprehensive narrative review of published literature was conducted. Articles describing paediatric populations were prioritised, but adult and animal studies were also considered where appropriate.

**Results:**

There is a dearth of high-level evidence supporting the use of ACT in acutely ill infants and children. Conversely, studies have highlighted the lack of effect of different modalities for a variety of conditions, and in some cases serious associated complications have been reported.

Airway clearance therapy may be considered when there is retention of pulmonary secretions, and the consequential airway obstruction impacts either acutely on respiratory mechanics and gaseous exchange and/or has the potential for long-term adverse *sequelae* [a condition that is the consequence of a previous disease or injury]. However, it should not be considered a routine intervention.

**Conclusion:**

Airway clearance therapy should not be performed routinely in children admitted to hospital with acute respiratory conditions. Patients should be clinically assessed and treatment planned according to individual presentation, in those with signs and symptoms that are potentially amenable to ACT.

**Clinical implications:**

This review can serve as a guide for physiotherapists in the respiratory management of children with acute respiratory illness, as well as identifying areas for clinical research.

## Introduction

‘Make a habit of two things: to help, or at least to do no harm’ – Hippocrates, ca. 400 BC

In South Africa, and elsewhere in the world, lower respiratory tract infections (LRTIs) constitute a major burden of disease and are one of the most common reasons for hospital admission among infants and children (Nair et al. 2013:1380–1390; Pediatric Global Burden of Disease 2016:267–287; Zar & Ferkol 2014:430–434). Paediatric patients admitted to hospital with acute LRTI are frequently referred for physiotherapy assessment and cardiopulmonary management.

This narrative, state-of-the-art review aims to critically synthesise published evidence, expert opinion and pathophysiological principles to describe the background and rationale, indications, effects, precautions or contraindications and application of commonly used airway clearance therapy (ACT) modalities for managing infants and children with acute lower respiratory tract illness.

## Methods

A non-systematic narrative review of published literature was conducted, using the following online search engines and databases: PubMed/Medline (https://www.ncbi.nlm.nih.gov/pubmed/); Physiotherapy Evidence Database (PEDro) (https://www.pedro.org.au/); EBSCOhost Research Platform (http://web.b.ebscohost.com/); Cochrane Database of Systematic Reviews (https://www.cochranelibrary.com/cdsr/reviews) and the National Institute for Health and Care Excellence (NICE) guidelines (https://www.nice.org.uk). In addition, the reference list of identified articles was scanned for potentially relevant articles.

Articles in the English language, with no limitations on date of publication or study design, were considered for inclusion if they described any aspect relevant to ACT in acute paediatric pulmonary disease. For the purposes of this review, only manual ACTs and those performed independently or with minimal assistance were included, while mechanical ACTs (such as high frequency chest wall oscillation, intrapulmonary percussive ventilation and mechanical insufflation–exsufflation) were not discussed. Articles describing paediatric populations managed with acute lower respiratory tract disease were prioritised, but adult and animal studies were also considered where there was insufficient paediatric evidence. Studies on children with chronic pulmonary disease were also considered for inclusion if they were relevant, and in the absence of sufficient condition-specific evidence.

Search terms used included general intervention terms such as ‘chest physiotherapy’, ‘chest physical therapy’, ‘airway clearance techniques’ and ‘airway clearance therapy’, as well as specific modalities, including ‘percussion’, ‘manual vibration’, ‘postural drainage’; ‘autogenic drainage’; ‘positive expiratory pressure therapy’; and ‘active cycle of breathing technique’. Population search terms were ‘children’, ‘infants’ and ‘paediatric’, and the condition-specific terms included general terms such as ‘lower respiratory tract infections’, ‘lung disease’ and ‘pulmonary infections’, and specific conditions, including ‘pneumonia’, ‘bronchiolitis’, ‘asthma’ and ‘atelectasis’.

### Ethical considerations

This article does not involve human subject research and therefore does not require ethical review board approval. This article followed all ethical standards for research without direct contact with human or animal subjects.

## Background – Normal and abnormal secretion clearance

In healthy children, the normal mucociliary clearance mechanism is responsible for maintaining patent airways, by moving pulmonary secretions in a cephalad direction, through coordinated ciliary beating, in conjunction with the expiratory airflow bias caused by dynamic airway compression during exhalation. This mechanism is analogous to an escalator (the so-called ‘mucociliary escalator’), moving debris upwards towards the mouth (De Boeck et al. 2008:607–612; Fink 2007:1210–1221; Hess 2001:1276–1293; Volsko 2013:1669–1678).

The vast majority of children admitted with acute LRTI, with normal muscle strength, mucociliary and chest wall function, will gain complete recovery following an acute LRTI, and receiving ACT as an adjunctive treatment is therefore unlikely to benefit the child in the longer term, while adding substantial financial cost (De Boeck et al. 2008:607–612) and potentially exacerbating the child’s condition. However, similar to a real escalator, in some children the system becomes overloaded during LRTI and the normal pulmonary defence system fails. Such failure of mucociliary clearance may occur because of excessive mucus production or a change in mucus viscosity or osmolality, or the system may be impaired by airway inflammation (Hess 2001:1276–1293).

Central secretions are usually cleared by coughing, which is a normal protective mechanism, but the cough may become ineffective and tiring for a child, particularly when it is out of proportion to the secretions that can be cleared (De Boeck et al. 2008:607–612). A cough can only clear to the sixth or seventh bronchial generations; secretions retained beyond that point will not be cleared by coughing alone (Frownfelter & Massery 2006:363–376). In addition, the high pressures and airflow during a cough may cause airway compression and actually lead to distal trapping of both air and secretions (Button & Button 2013; Fink 2007:1210–1221). In these settings, ACT may be appropriate to facilitate clearance of the retained obstructive pulmonary secretions (Volsko 2013:1669–1678), thereby reducing airway resistance and improving work of breathing and gaseous exchange in the short term (De Boeck et al. 2008:607–612). In addition, ACT in selected children with acute respiratory disease may facilitate early weaning from mechanical ventilation; prevent further respiratory complications (such as atelectasis and bronchiectasis); re-expand collapsed lung segments or lobes and hasten recovery (Ciesla 1996:609–625; Hess 2001:1276–1293; Main et al. 2004:1144–1151; Ntoumenopoulos 1997:292–293; Oberwaldner 2000:196–204; Wallis & Prasad 1999:393–397).

## Airway clearance techniques

Various modalities are commonly used by physiotherapists to mobilise and facilitate clearance of pulmonary secretions in infants and children; however, very few are supported by high-level scientific evidence (De Boeck et al. 2008:607–612; Schechter 2007:1382–1390). The manual application of techniques such as percussions and vibrations, usually combined with gravity-assisted positioning (postural drainage [PD]), has traditionally been referred to as ‘conventional chest physiotherapy’ (De Boeck et al. 2008:607–612). However, the ACT ‘toolbox’ has a number of different therapeutic modalities to choose from when treating children with secretion encumbrance. In an effort to move towards a problem- and solution-based approach to cardiopulmonary rehabilitation, many physiotherapists now use the collective term ‘airway clearance therapy’ in preference to ‘chest physiotherapy’ (De Boeck et al. 2008:607–612), a shift which has been adopted by the author.

The evidence for ACT in acute paediatric LRTI is extremely limited (De Boeck et al. 2008:607–612), with existing studies limited by small sample sizes, research design and lack of standardisation or clear documentation of the intervention (Argent & Morrow 2012:238–239; De Boeck et al. 2008:607–612; Hess 2001:1276–1293). It is alarming that many of the studies that have been conducted suggest that conventional chest physiotherapy, which is probably still the most commonly practised ACT, may be either useless or frankly harmful in a number of paediatric conditions (Button et al. 1997:148–150, 1998:330–334, 2004:435–439; Chalumeau et al. 2002:644–647; Chaneliere et al. 2006:1410–1412; Harding et al. 1998:440–444; Krause & Hoehn 2000:1648–1651; Reines et al. 1982:451–455; Wallis & Prasad 1999:393–397; Weissman et al. 1984:815–818; Zidulka et al. 1989:2833–2838), and the objective evidence for any benefit of ACT is similarly lacking (Hess 2001:1276–1293). Complications attributed to conventional chest physiotherapy and endotracheal suctioning in infants and children include changes in blood pressure, cardiac arrhythmia, raised intracranial pressure and decreased cerebral oxygenation, hypoxia, increased metabolic demand and oxygen consumption, gastro-oesophageal reflux, pneumothorax, atelectasis and even death (Argent & Morrow 2004:1014–1016; Asher et al. 1990:146–151; Button et al. 2004:435–439; Chalumeau et al. 2002:644–647; Chaneliere et al. 2006:1410–1412; Harding et al. 1998:440–444; Oberwaldner 2000:196–204; Reines et al. 1982:451–455; Wallis & Prasad 1999:393–397; Zidulka et al. 1989:2833–2838). It is not clear if the newer active ACT modalities afford better safety profiles than conventional chest physiotherapy techniques (De Boeck et al. 2008:607–612).

Absence of evidence supporting the efficacy of ACT in acute paediatric respiratory disease does not, however, equate to ‘evidence of absence’ of effect (De Boeck et al. 2008:607–612). When interrogating some clinical studies that have overall shown no benefit of ACT, it is interesting that there is usually a subgroup of patients who clearly derive benefit, a group in which ACT causes harm or worsening outcomes (Argent & Morrow 2012:238–239), and another group in which ACT has no effect at all (Main et al. 2004:1144–1151; Main & Stocks 2004:1152–1159; Morrow, Futter & Argent 2006:121–126). In a randomised cross-over trial comparing non-standardised ‘chest physiotherapy’ and endotracheal suction to endotracheal suction alone, in sedated, mechanically ventilated infants and children, the chest physiotherapy group showed higher compliance, tidal volume and alveolar dead-space, with no change in gas exchange, from blood gas analysis (Argent & Morrow 2004:1014–1016; Main et al. 2004:1144–1151; Main & Stocks 2004:1152–1159). Notably, though, up to a third of patients in both groups deteriorated following the intervention, and the authors were unable to identify groups who were more or less likely to respond to physiotherapy (Argent & Morrow 2004:1014–1016; Main et al. 2004:1144–1151; Main & Stocks 2004:1152–1159).

No single ACT modality has ever been convincingly shown to be superior to another, although for an individual one ACT may be better (or more harmful) than others (Volsko 2013:1669–1178). In the absence of clear evidence, it is therefore our challenge as physiotherapists to use clinical reasoning (based on sound pathophysiological principles) to decide, firstly, whether or not *an individual patient* is likely to benefit from ACT; to determine whether there are any factors placing the patient at risk of harm if ACT were administered (De Boeck et al. 2008:607–612); and then, if considered indicated, to decide which modality or modalities should be applied for the greatest benefit, and with the least potential for harm. The prescription of ACT should always be individualised (Volsko 2013:1669–1678), and patient-specific factors should be considered, including age, patient preference, disease condition, acuity of illness, developmental level, propensity of specific contraindications and so on.

### Airway clearance therapy modalities

**FIGURE 1:**
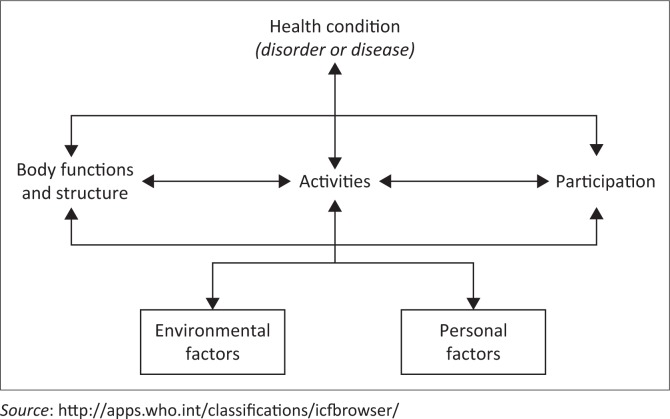
The International Classification of Functioning, Disability and Health model (World Health Organisation).

Although a number of specific ACT modalities are described, it is the author’s opinion that the approach to any physiotherapy intervention in children should be broad, with attention paid to the holistic multi-system care of children who may present with complex disease processes. To this end, clinical assessment using the World Health Organisation’s International Classification of Functioning, Disability and Health (ICF) should be implemented to identify the many contributing factors to functional and disease states (Figure 1) (Cerniauskaite et al. 2011:281–309). For example, a child with severe LRTI (health condition) may have impairments in the domains of body function and structure (e.g. secretion retention and airway inflammation), activity (e.g. walking a certain distance or developmental milestone regression), participation (e.g. involvement in school sports and playing with other children), environment (e.g. home exposure to environmental air pollutants contributing to poor health) and personal factors (e.g. fear of hospital environment, needle phobia or separation anxiety), all of which could be considered when developing an holistic management plan.

**FIGURE 2:**
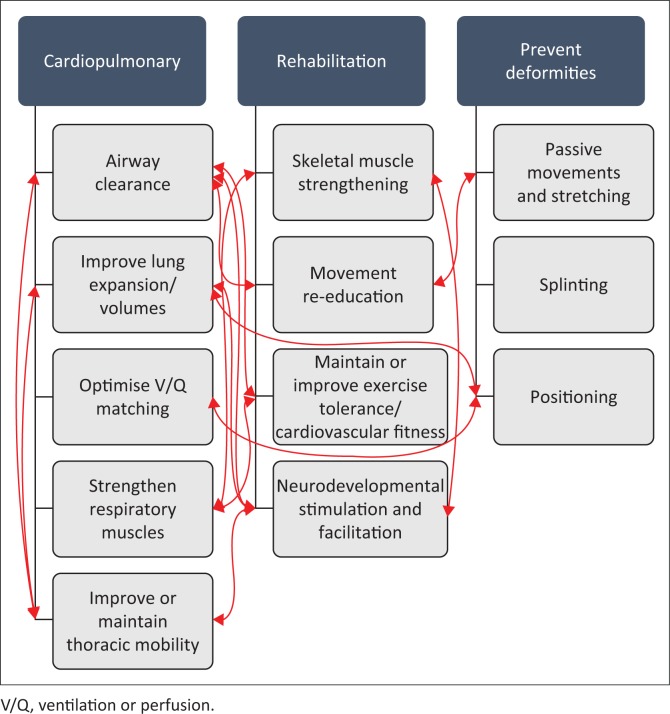
Selected examples of the inter-relationships among different systems and physiotherapeutic interventions in infants and children.

The awareness that all systems are inter-related is, in the author’s opinion, essential in planning appropriate ACT for children (Figure 2). For example, by positioning a child with cerebral palsy and LRTI to normalise tone, promote functional movement and maintain range of motion, one may also be using gravity to mobilise secretions centrally and, by influencing ventilation distribution and clearing obstructive secretions, one might also improve regional lung expansion and prevent aspiration and further respiratory sequelae. Therefore, it is recommended that ACT modalities should not be applied in isolation, but rather in combination with general rehabilitation, developmental stimulation and other supportive care. With that caveat, however, common ACT modalities will be discussed below.

#### Positioning for secretion mobilisation

Therapeutic positioning aims to enhance mucociliary secretion clearance while optimising ventilation/perfusion (V/Q) matching (Lupton-Smith et al. 2014:764–771), thereby reducing the work of breathing (Clini & Ambrosino 2005:1096–1104; Stiller 2000:1801–1813). William Ewart first introduced the concept of PD in 1901 (based on *in vitro* studies using bronchial casts), which he referred to as the ‘empty bronchus treatment by posture’ for children with bronchiectasis (Ewart 1901:70–72). Subsequently, Nelson (1934) described a small *in vivo* case series (*n* = 3), using iodised oil-instilled bronchograms (Nelson 1934:251–255), and in the 1940s Foster-Carter conducted further *in vitro* work using bronchial casts (Foster-Carter 1943:451–456). These studies led to the development of a number of standardised ‘postural drainage’ positions for the clearance of specific segments of the lung. Some of the positions advocated involved tipping the patient into an inverted, head-down position. Both Ewart (1901) and Nelson (1934) advocated maintaining PD positions for up to hours at a time to enable ‘continuous drainage’ of the lungs. Since the early 20th century, there has been very little modification of the prescribed PD positions, and this practice continues to be recommended in the majority of cardiopulmonary physiotherapy textbooks, despite very little supportive scientific evidence (Hess 2001:1276–1293).

A small cross-over study of patients with cystic fibrosis showed no significant benefit of PD above other airway clearance methods (Lannefors & Wollmer 1992:748–753), as had been reported previously (Oldenburg et al. 1979:739–745). Lannefors and Wollmer (1992) also reported that seven of their nine participants drained more from the left, dependent lung, when lying in the left decubitus PD position (Lannefors & Wollmer 1992:748–753), calling into question the principle of PD as a gravity-dependent clearance technique. Lannefors and Wollmer’s findings (1992) suggest that the effects of positioning on secretion clearance may relate more to gravity-dependent changes in regional ventilation (with increased regional volume and greater expiratory flow) than secretion drainage by gravity alone (Button & Button 2013:8). Although preferential ventilation in spontaneously breathing adults is always to the dependent lung, in children the pattern of ventilation in response to positional changes is highly variable (Lupton-Smith et al. 2014:764–771), again reinforcing the need for individual prescription of intervention rather than using a standardised approach (Button & Button 2013). In contrast to the study described above (Lannefors & Wollmer 1992:748–753), Berney, Denehy and Pretto (2004) reported that peak expiratory flow rates and sputum production were significantly improved with manual hyperinflation in the head-down tilt position compared to manual hyperinflation in the flat side-lying position, in a randomised cross-over trial of intubated and mechanically ventilated adults (Berney et al. 2004:9–14). No studies investigating PD in the management of children with acute pulmonary disease were identified.

In addition to lack of high-level evidence, the value of the standard PD positions is pathophysiologically unsupported. The analogy of a ketchup (tomato sauce) bottle is useful in this context – when you open a new bottle, even when held upside down, there is usually no movement of the sauce at all until you hit the bottom of the bottle. As with the ‘ketchup model’, viscous secretions, as would occur during LRTI, are unlikely to move because of gravity alone – one first has to lower the viscosity of the mucus for it to flow. In the physiotherapy context, some manual techniques (such as percussions and vibrations) may reduce viscosity of secretions, enabling this gravity-dependent flow, but PD alone is unlikely to be beneficial. Furthermore, there are clear reports of harm arising from head-down PD positioning in children and infants (Button & Button 2013). Inverted positioning may increase gastro-oesophageal reflux (Button et al. 1998:330–334, 2003:208–213, 2004:435–439; Vandenplas et al. 1991:23–26) and intracranial pressure (Emery & Peabody 1983:950–953); place the diaphragm at a mechanical disadvantage leading to the potential for respiratory failure in infants whose primary muscle of inspiration is the diaphragm (Vivian-Beresford, King & MaCauley 1987:184–190); reduce functional residual capacity (Nunn 1993) and increase venous return, thereby increasing the work of the heart. Conversely, the upright position improves end-expiratory lung volumes (maintaining functional residual capacity above closing capacity and thereby preventing airway closure) and oxygenation, and may protect against ventilator-associated pneumonia in the paediatric intensive care unit (PICU) context (Dellagrammaticas et al. 1991:429–432; Drakulovic et al. 1999:1851–1858; Nunn 1993; Stark et al. 1984:64–71). In children, where functional residual capacity is very close to closing capacity, it is particularly important to maintain functional residual capacity well above closing capacity, to keep the lungs open and optimally ventilated (Nunn 1993).

As Fink (2007) observed, gravity is not the primary mechanism for mucus transport in the lung – if it were, then there would be a tendency for secretions to pool in dependent parts of the lung (Fink 2007:1210–1221). The cephalad airflow bias of the mucociliary escalator instead facilitates transport of mucus against gravity towards the head. There are a number of ACT modalities other than PD, which support and facilitate this normal cephalad mucus transport mechanism, thereby offering a more convenient, and possibly more effective means of mobilising secretions than attempting to use gravity alone (Fink 2007:1210–1221).

Considering the lack of supporting evidence and the potential for adverse events, it is therefore recommended that the inverted position should not be used in paediatric practice. In the author’s opinion, positions should be chosen according to individual indication and effect on secretion clearance, rather than using a ‘recipe’ approach for prescription of therapy. Positions chosen to facilitate secretion clearance in children could include side-lying, upright sitting and prone (and variations of these), preferably with the head of the bed raised to optimise lung volumes (Nunn 1993). It may be that change in position (i.e. mobilisation) is more beneficial in terms of secretion clearance than sedentary positioning for extended periods. This requires further research.

#### Mobilisation and active exercise

In addition to facilitating secretion clearance, mobilisation and active exercise aim to improve thoracic mobility; increase lung volume; improve or maintain cardiovascular fitness, exercise tolerance and muscle strength; prevent postural deformities; improve bone ossification, bladder and bowel function; maintain skin integrity and confer psychological benefits (Button & Button 2013; Morrow 2015:174–181). Mobilisation techniques and exercise prescription should be selected according to the individual patients’ general condition, chronological (or corrected) age and developmental level (Morrow 2015:174–181). The term ‘mobilisation’ includes a range of active, passive or assisted techniques, including limb exercises, bed mobility, sitting out of bed, standing, crawling and walking. ‘Exercise’ extends mobilisation to activities enhancing strength and/or endurance (Button & Button 2013). It is recommended that mobilisation be implemented early in the course of acute illness, even for critically ill children admitted to the intensive care unit, to prevent the development of critical illness and immobility-related morbidity, such as muscle weakness, positional atelectasis, skin ulcers and positional deformities, and to improve functional outcomes (Choong et al. 2017). There is some evidence in patients with cystic fibrosis that exercise, as an adjunct to other ACT modalities, improves secretion clearance (Mcllwaine 2007:8–16; Thomas, Cook & Brooks 1995:846–850); however, there is no research into the benefits of exercise for secretion clearance in infants and children with acute LRTI. It is generally recommended that intense physical exercise should be avoided in children with acute pneumonia because of the risk of cardiovascular complications (Durakovic et al. 2009:387–390). In addition, caution should be taken in children with a high fever, pulmonary hypertension and exercise-induced bronchospasm (Button & Button 2013). However, active play as aerobic exercise (within patient tolerance) may be a useful adjunctive technique for airway clearance in children with acute LRTI, which may be better tolerated and enjoyed by young children than sedentary manual techniques. This is an area for future research.

#### Chest manipulations or manual chest physiotherapy

Percussion and vibrations are commonly used ACT modalities, applied manually or mechanically. The principle behind these techniques relates to the properties of respiratory mucus as a non-Newtonian and thixotrophic gel, which is between that of an elastic solid and a viscous liquid (Lai et al. 2009:86–100). Thixotrophic gels are highly viscous under static conditions, but become less viscous and able to flow when shaken or agitated (Lai et al. 2009:86–100). It is postulated that by applying percussion or vibrations to the chest wall, mechanical energy is transmitted into the airways, thereby reducing the viscosity of bronchial secretions, which can then be more easily cleared by positioning, cough or suctioning.

Manual vibration, with a combination of compression and oscillation, has been shown to increase expiratory flow rate via increased intrapleural pressure in mechanically ventilated children in PICU (Gregson et al. 2012:e97–e102), suggesting potential benefit in secretion clearance. Manual techniques may be useful in specific circumstances and disease conditions, but in many cases they are useless, or even harmful (Wallis & Prasad 1999:393–397). In critically ill adults, percussion has been associated with cardiac arrhythmia and a decrease in pulmonary compliance (Stiller 2000:1801–1813), and it has been suggested that both percussion and vibrations may cause or exacerbate bronchospasm (Kirilloff et al. 1985:436–444). In acutely ill children, any potential benefits of intervention must be carefully balanced against risk of harm before they are implemented (Morrow 2015:174–181). From personal experience, some children enjoy percussion and tolerate it well, while in others there may be a perception of the perpetuation of physical abuse (hitting), a problem that is rife in our community.

The use of any external percussion or vibration method is currently not supported by high-level scientific evidence (Branson 2007:1328–1342; Hess 2001:1276–1293; Kirilloff et al. 1985:436–444; Krause & Hoehn 2000:1648–1651; Van Der Schans et al. 1999:1477–1486), and research is urgently needed to determine efficacy and safety in different contexts. Percussion and vibrations are contraindicated in children with severe osteoporosis and frank haemoptysis, and precaution should be taken in those with rib fractures, hyper-reactive airways and bronchospasm (Button & Button 2013).

#### Breathing exercises

A number of different breathing exercises are used as ACT modalities, including deep and localised breathing exercises (thoracic expansion exercises), positive expiratory pressure (PEP) and oscillatory PEP therapy, the active cycle of breathing technique (ACBT), autogenic drainage and the forced expiratory technique (FET) (Morrow 2015:174–181). Most of these techniques were developed for and tested in children and adults with cystic fibrosis and other chronic sputum-producing illnesses, and there is limited evidence for their use in acute conditions (Lewis, Williams & Olds 2012:155–172). However, it makes physiological sense that where there are obstructive secretions, techniques aiming to increase expiratory flow and promote secretion clearance may be effective. These techniques can be used in any position, may be done independently (by older children and adolescents) and may also be combined with other techniques such as positioning and vibrations. De Boeck and Zinman (1984) observed that deep breathing exercises were among the safest, most effective and cheapest strategies for keeping the lungs expanded and secretions moving (De Boeck & Zinman 1984:182–184).

**FIGURE 3:**
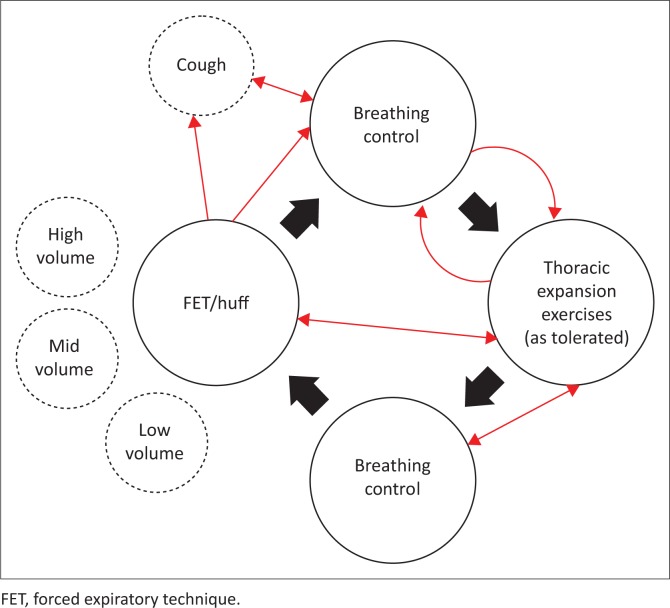
Components and examples of variations in the active cycle of breathing technique.

**Active cycle of breathing technique:** The ACBT comprises several deep breaths or thoracic expansion exercises and FET or ‘huff’, interspersed with episodes of breathing control (relaxed diaphragmatic breathing) (Lewis et al. 2012:155–172). It has been suggested that there should not be a ‘one-size-fits-all’ approach to ACBT and FET, as the most effective technique will vary among different patients, and even for the same patient under different circumstances (Lewis et al. 2012:155–172). Some patients will require several periods of breathing control, with a limited number of deep thoracic expansion breaths and FETs, particularly if they have severe lung disease with shortness of breath (Figure 3).

A systematic review and meta-analysis showed that ACBT seems to have a greater beneficial short-term effect on secretion clearance than conventional chest physiotherapy, external oscillatory devices and a control group (Lewis et al. 2012:155–172).

It is postulated that increasing inspiratory volumes during thoracic expansion exercises may recruit collateral ventilatory channels, thereby enhancing expiratory flow behind retained secretions. This effect may be enhanced by adding an inspiratory hold, to allow sufficient time for obstructed lung units to fill and to improve homogeneity of inflation (Button & Button 2013). In young children under 3 years of age, however, the collateral ventilatory channels are poorly developed (Cetti, Moore & Geddes 2006:371–373; Rogers & Doull 2005:233–238; Rosenberg & Lyons 1979:125–134); therefore, this mechanism cannot explain the observed benefit of ACBT in this population (Schechter 2007:1382–1390).

**FIGURE 4:**
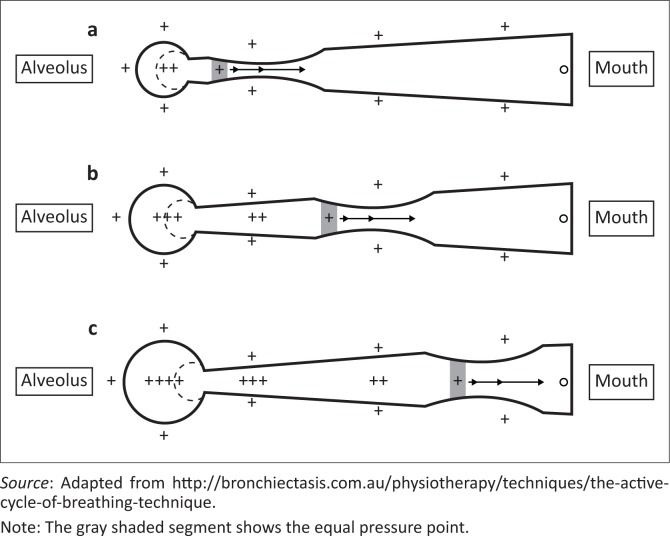
The equal pressure point during a forced expiratory technique at different lung volumes, (a) low volume, (b) mid volume and (c) high volume.

The secretions mobilised during the thoracic expansion exercise component of ACBT are moved downstream (towards the mouth) during the FET. The principle behind the FET relates largely to manipulation of thoracic pressures and airway dynamics. At the start of forced exhalation, the pressure inside the lungs decreases from the peripheral airways to the mouth, and there is a point where the pressure in the airways is the same as outside the airways (equal pressure point; Figure 4). Downstream from the equal pressure point, the pressure outside is greater than inside the airways, leading to a point of narrowing of the airways. According to Bernoulli’s principle, airflow accelerates through a narrowing within a tube (associated with a decrease in pressure), and this acceleration and turbulent airflow causes shear forces which help to remove mucus from the mucosal walls of the airways (Button & Button 2013). During a FET, a wave of equal pressure points effectively moves peripherally into the airways as lung volume decreases and the pressure in the airway drops. The position of the equal pressure point depends on the lung volume and the pressure differential between the outside and inside of the airways (Button & Button 2013; Rogers & Doull 2005:233–238).

Therefore, by changing the volume of the FET, one could manipulate where the equal pressure point occurs in the airway (Rogers & Doull 2005:233–238), thereby directing secretion mobilisation to more peripheral airways (at low lung volumes), medium-sized airways (at mid-lung volumes) and finally to large central airways (at high lung volumes) (Button & Button 2013). It is the author’s opinion that this approach is appropriate when determining the FET technique, depending on where secretions are being retained in the respiratory tree (Figure 4). A FET or ‘huff’ should be done using mild-to-moderate force, with the glottis open and the initial inspiration and duration of exhalation adjusted to optimise clearance of secretions (Van Der Schans 1997:367–370).

Active cycle of breathing technique may be taught to children from as early as 2 years of age (under supervision), and can be performed independently from about 8 years of age (Fink 2007:1210–1221). In the author’s experience, fun blowing games can initially be used to teach and facilitate the different components of ACBT in very young children. For example, blowing a windmill will require deep inspiration (thoracic expansion exercises); and huffs can be taught using a mirror, which is misted up during the manoeuvre. In the author’s opinion, breathing control never needs to be taught, but the child should be positioned appropriately such that their pattern of breathing is normalised as far as possible during rest periods.

**Positive expiratory pressure therapy:** Positive expiratory pressure therapy involves breathing out against an expiratory resistance (Olsen, Lannefors & Westerdahl 2015:297–307), and aims to optimise secretion clearance, improve functional residual capacity and tidal volume and reduce hyperinflation or air trapping (Olsen et al. 2015:297–307). Positive expiratory pressure effectively splints the airways during exhalation, thereby promoting more homogenous expiratory flow (Rogers & Doull 2005:233–238). In addition, in older children with developed collateral channels, PEP therapy may enable more air to enter the airways via collateral ventilation channels, to behind the secretions. This theoretically builds up pressure behind the secretions which facilitates secretion mobilisation into larger, central airways (Rogers & Doull 2005:233–238).

A number of manufactured PEP devices exist (Olsen et al. 2015:297–307), at various financial costs and with variable availability in different settings, including PEP masks and valves. Although most studies have been conducted using PEP masks, there is no evidence that other techniques or devices are inferior. Positive expiratory pressure therapy has even been described in infants, using a mask held over the infant’s mouth and nose (Button & Button 2013).

Positive expiratory pressure therapy for airway clearance is usually combined with components of the ACBT, but the thoracic expansion exercises are variably performed using tidal, slightly above tidal or thoracic expansion breaths; and expiration (active but not forced) is done against PEP (Olsen et al. 2015:297–307). Airways are ‘opened’ to allow air to flow behind obstructive secretions, whereupon they can be cleared by FET without applied PEP (Button & Button 2013; Olsen et al. 2015:297–307) (allowing full utilisation of the equal pressure point theory). However, in patients with very unstable airways, the FET may also be done against resistance (high pressure PEP), to prevent excessive dynamic compression of the airways and promote homogenous lung emptying, thereby avoiding further gas trapping (Oberwaldner, Evans & Zach 1986:358–367; Olsen et al. 2015:297–307).

**FIGURE 5:**
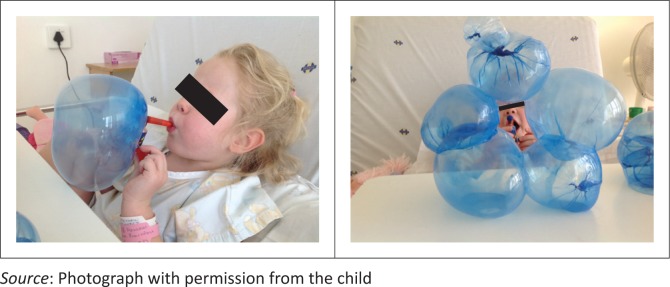
The author’s then 4-year old daughter doing ‘fun’ positive expiratory pressure therapy following Nissen’s funduplication surgery and postoperative pulmonary complications.

For children, fun PEP therapy, using blowing games such as bubbles, windmills and whistle blowing, can be used. Although PEP is not well controlled or measured using such toys, their use could optimise patient compliance and enjoyment of their ACT (Figure 5), but this requires confirmation in clinical studies.

**Oscillating positive expiratory pressure therapy:** Oscillating PEP therapy involves a combination of PEP and high-frequency expiratory flow oscillation, with the aim of clearing secretions and reducing air trapping. Devices include Flutter^®^ valves, Acapella^®^ devices, RC-Cornets^®^, Shaker^®^ devices and Bronch-u-Vibe^®^ devices (Olsen et al. 2015:297–307). Bottle PEP is also a form of oscillatory PEP, as the bubbles created and burst during expiration through the water, provide oscillatory resistance to airflow. The oscillatory vibration of the airway wall reduces the visco-elasticity of the thixotrophic mucus in the airways, while PEP holds airways open and enhanced expiratory flow moves secretions towards the mouth (Button & Button 2013). The Flutter^®^ device is the only physiotherapy technique that has objectively been demonstrated to change mucus rheology (App et al. 1998:171–177). The method for using oscillatory PEP devices is similar to that of PEP devices – patients are instructed to take a deeper than normal breath in; they may optionally hold the inspiratory breath for a few seconds and then exhale actively through the device (to a low functional residual capacity level, but not completely to residual volume) (Olsen et al. 2015:297–307). Active breaths can be interspersed with breathing control, as for the ACBT, as well as FET and coughing as needed to clear mobilised secretions. For children particularly, assistance may be needed to stabilise the cheeks during exhalation, so oscillations are transmitted optimally to the lungs (Olsen et al. 2015:297–307). The number of breaths per cycle and the number of cycles per session should be individualised.

Bottle PEP may be used in children as soon as they can coordinate blowing (and not sucking). Additional incentive may be added by including some dishwashing detergent and/or food colouring so that soapy bubbles are created during expiration. The concerns about bottle PEP for children admitted to hospital relate to infection control, as the water could be a source of contamination with hospital-acquired organisms, thereby increasing the risk of nosocomial infection. If bottle PEP is to be used, the bottle and tubing must be washed and dried after each use, and should never just be left next to the patient’s bed (Rogers & Doull 2005:233–238).

Bottle PEP devices can be made using a bottle and wide-bore tubing (> 8 mm internal diameter [Mestriner et al. 2009:504–508]), with the bottom of the tubing resting against the base of the bottle, and water filled to the required depth according to the PEP required (usually 5–10 cm H_2_O), thereby creating an underwater seal. The bottle should not be sealed at the top, as there needs to be an adequate air-escape orifice to prevent build-up of pressure above the water-column pressure (Mestriner et al. 2009:504–508).

Contraindications to any PEP therapy are undrained pneumothorax and frank haemoptysis (Button & Button 2013). Precautions should also be taken in patients with any of the following: drained pneumothorax, because of risk of air leak; after lung lobectomy or after lung transplantation, as it may cause pneumothorax or compromise the anastomosis; haemodynamic instability; undrained empyema or lung abscess (because of the risk of sudden release of a large volume of loculated fluid); inability to tolerate therapy because of increased work of breathing; middle ear infection (because of the risk of increasing pressure in the Eustachian tubes) and portal hypertension with oesophageal varices (owing to the risk of precipitating variceal bleeds) (American Association for Respiratory Care 1993:516–521; Button & Button 2013).

**Autogenic drainage:** Autogenic drainage is a self-drainage technique, which aims to generate the highest possible expiratory airflow, at different lung volumes and without causing dynamic airway collapse, to move secretions centrally (Button & Button 2013). Controlled breathing techniques are used to maximise expiratory flow while minimising airway closure (Fink 2007:1210–1221). The patient initially takes a deeper than normal breath in, then breathes out (actively but not forced, and with an open glottis) to expiratory reserve volume, and then takes tidal breaths at low volume until the secretions are heard or felt on the breath. The cough should be suppressed and the lung volume increased to mid volumes for a further series of breaths until secretions are again heard or felt. Thereafter the patient takes larger (into inspiratory reserve volume) breaths and may then huff or cough to clear the secretions (Agostini & Knowles 2007:157–163). This technique may be effective but requires concentration, and is difficult to teach to young children (Fink 2007:1210–1221). Active autogenic drainage may, therefore, be more appropriate in older children with chronic lung disease rather than those with acute LRTI. This warrants clinical research.

Autogenic drainage may be applied in a passive form (assisted autogenic drainage) to infants and young children, by applying external compression to the chest wall, thereby manipulating lung volume and increasing expiratory flow. Assisted autogenic drainage may be performed with the infant on the physiotherapists’ lap, bouncing on a therapy ball, which may encourage relaxation of the child and improve expiratory airflow (Lee, Button & Tannenbaum 2017:2). A pilot randomised controlled trial (*n* = 29) (Corten et al. 2018) to determine the effect of assisted autogenic drainage in young children admitted to hospital with uncomplicated pneumonia, compared to standard nursing care alone (Corten et al. 2018), reported there was a trend towards a shorter time to discharge in the intervention group (*p* = 0.06), but no other significant benefit of assisted autogenic drainage. Importantly, though, no adverse events occurred, suggesting that this technique could be added to our ‘ACT toolbox’ for use in infants and children, and this warrants further investigation (Corten et al. 2018).

#### Manual hyperinflation

In adult intensive care units, manual hyperinflation is a common technique used to expand the lung and mobilise secretions (McCarren & Chow 1996:203–208; Patman, Jenkins & Stiller 2000:157–171), and it is also used in some centres around the world for the treatment of ventilated children and infants (De Godoy, Zanetti & Johnston 2013:258–262; McCord et al. 2013:374–377). Manual hyperinflation is usually performed by applying a series of deep manual inflations with brief inspiratory holds, followed by a rapid release of the bag to enhance expiratory flow and mimic a cough (Stiller 2000:1801–1813).

There are conflicting reports on the efficacy and safety of manual hyperinflation (Barker & Adams 2002:157–169; Choi & Jones 2005:25–30; Patman et al. 2000:157–171; Stiller et al. 1990:1336–1340), but the risk of baro- or volutrauma and subsequent lung injury is a particular concern in children (Carpenter 2004:231–237; Dreyfuss & Saumon 1998:294–323; Morrow 2015:174–181; O’Donnell, Davis & Morley 2003:76–82). A systematic review (De Godoy, Zanetti & Johnston 2013:258–262) of manual hyperinflation in children could only include three studies (Gregson et al. 2007:1017–1028, 2012:e97–e102), two of which were observational and one was a randomised cross-over study (Main et al. 2004:1144–1151), which did not evaluate the impact of manual hyperinflation independent from other ACT modalities.

Considering the lack of evidence supporting manual hyperinflation in critically ill infants and children, and the potential for harm, I have recommended that this practice should not be considered an acceptable component of standard ACT for ventilated infants and children (Morrow 2015:174–181). However, if secretions cannot be cleared using standard ACT techniques, physiotherapists may consider adding manual inflations with brief inspiratory hold and rapid release, using an open-ended bag (e.g. Mapleson C circuit) in order to facilitate expiratory flow and proximal secretion mobilisation. I would prefer using the term ‘normo’-inflation, owing to the risks of volu- and barotrauma. If manual inflations are performed, positive end-expiratory pressure (PEEP) should be maintained throughout, and the applied peak inspiratory pressure limited to < 5 cm H_2_O above the peak inspired ventilator pressures (using a pressure manometer).

#### Endotracheal suctioning

Although not strictly a physiotherapy ACT modality in itself, suctioning may be necessary after mobilising secretions using the aforementioned techniques, in the face of a child with an ineffective cough and/or in those with an artificial airway. Recommendations and clinical guidelines for endotracheal suctioning of intubated patients have been published previously, and are not the focus of this review (Morrow & Argent 2008:465–477). In non-intubated patients, suctioning can be performed through the nasopharyngeal or oropharyngeal route, with the oral route mandatory in the case of a base of skull fracture or severe epistaxis. It is important to note that suctioning alone may cause significant complications, including hypoxia, mucosal trauma, pneumothorax, atelectasis, raised intracranial pressure, cardiac arrhythmia, pain and discomfort (Morrow & Argent 2008:465–477). Care should be taken to ameliorate the risk of these complications when suctioning patients.

### Indications for airway clearance therapy in acute paediatric lower respiratory tract infections

Indications or contraindications for or against chest physiotherapy should never be formulated on the basis of diagnostic entities but should rather stem from a detailed analysis of the prevailing individual pathophysiology. (Oberwaldner 2000:196–204)

Airway clearance therapy is seldom indicated in a previously well child who presents with acute LRTI and uncompromised mucociliary clearance, fully mobile and able to cough and clear their secretions effectively and without undue fatigue (De Boeck et al. 2008:607–612). Considering that the main aim of ACT is to remove obstructive secretions to prevent or mitigate the mechanical consequences of airway obstruction, only children with retention of secretions are potentially likely to benefit from treatment (Schechter 2007:1382–1390; discussion 90–91). The child’s medical diagnosis should not be the deciding factor about whether or not ACT is performed. Each patient should be comprehensively clinically and radiologically assessed to determine whether their individual pathophysiology is potentially amenable to ACT intervention (Morrow 2015:174–181; Oberwaldner 2000:196–204). The concept of ‘routine’ CPT for children with acute respiratory disease has been deemed by many authors to be inappropriate, outdated and has the potential to cause physical, psychosocial and financial harm (De Boeck et al. 2008:607–612; Krause & Hoehn 2000:1648–1651; Walsh, Hood & Merritt 2011:1424–1444).

Despite a lack of high-level evidence, different ACT modalities are likely to be beneficial for the treatment of atelectasis caused by mucus plugging (Bilan, Galehgolab & Shoaran 2009:467–469; Branson 2007:1328–1342; Galvis, Reyes & Nelson 1994:326–330; Peroni & Boner 2000:274–278; Schechter 2007:1382–1390; Wong & Fok 2003:43–50) and for children admitted with acute-on-chronic conditions such as those with neuromuscular disease admitted with an acute respiratory exacerbation (Schechter 2007:1382–1390).

Manual ACTs have been shown, at best, to be of minimal to no benefit in acute asthma, where ACT modalities could exacerbate bronchospasm and increase oxygen demand (Asher et al. 1990:146–151; Hondras, Linde & Jones 2005:CD001002; Walsh et al. 2011:1424–1444).

A Cochrane systematic review of 12 randomised controlled trials, with >1200 participants in total (Roque i Figuls et al. 2016:CD004873), concluded that a number of ACT modalities (including vibration, percussion and FET) did not improve severity of disease, respiratory parameters, length of hospital stay or duration of oxygen requirements in infants admitted to hospital with uncomplicated bronchiolitis. Although slow passive expiratory techniques did not confer overall benefit, there was a suggestion that these techniques may provide transient relief in some cases (possibly through reduction in air trapping), and this warrants further investigation. Airway clearance therapy cannot, therefore, be considered as standard management in infants with uncomplicated bronchiolitis, and this recommendation appears in local and international guideline documents (Zar et al. 2016:27–29).

Systematic reviews have reported that there is insufficient data to show whether or not ACT is beneficial or harmful in a number of clinical outcomes in children with acute uncomplicated pneumonia (Chaves et al. 2013:CD010277; Corten, Jelsma & Morrow 2015:256). One of the two randomised controlled trials included in these reviews (Lukrafka et al. 2012:967–971) may have been underpowered to detect a 2-day *increase* in hospital length of stay in the intervention group, and the other study (Paludo et al. 2008:791–794) also reported a longer duration of coughing (*p* = 0.04) and adventitious sounds on auscultation (rhonchi) (*p* = 0.03) in those who received ACT compared to controls. This reinforces the suggestion that ACT should never be used routinely in children with acute LRTIs, but should be initiated on a clear indication following individual assessment.

In the PICU context, it is understood that mucociliary clearance is compromised in intubated patients, because of a combination of factors (Morrow 2015:174–181). Therefore, all intubated and mechanically ventilated infants and children will require endotracheal suctioning to maintain patency of their airways (Morrow & Argent 2008:465–477), but only a small proportion of these are likely to benefit from ACT (Argent et al. 2014:7–14). Considering the lack of evidence supporting the use of routine ACT in ventilated infants and children, as well as the potential for serious complications in this highly vulnerable (and often clinically unstable) population, manual ACT is not indicated routinely for ventilated infants and children (Krause & Hoehn 2000:1648–1651; Morrow 2015:174–181; Schechter 2007:1382–1390). Krause and Hoehn (2000) stated, ‘chest physiotherapy must be considered as the most stimulating and disturbing intensive care procedure in mechanically ventilated patients’ (pp. 1648–1651). It has therefore been suggested that, rather than focus on manual ACT interventions for critically ill infants and children, physiotherapists should engage, as part of the multidisciplinary PICU team, in appropriate holistic management such as pain control, positioning, lung protective ventilation, adequate humidification of ventilator gases, maintaining impeccable levels of hygiene and infection control, and PICU-based rehabilitation and early mobilisation (among others) – all these good care principles will likely benefit the child in terms of secretion management as well as optimising functional outcomes of critical illness (Morrow 2015:174–181).

Over-servicing is defined as:

the supply, provision, administration, use or prescription of any treatment or care … which is medically and clinically not indicated, unnecessary or inappropriate under the circumstances or which is not in accordance with the recognised treatment protocols and procedures, without due regard to both the financial and health interests of the patient. (Health Professionals Council of South Africa 2016:5)

Over-servicing is considered unethical by the Health Professionals Council of South Africa (2016) and, more importantly, may cause harm to a particularly vulnerable group of patients with limited autonomy. Therefore, regardless of diagnosis, it is essential that the prescription of ACT be very carefully considered for every patient, that the paediatric patient be reassessed at each contact and the prescription reviewed with changing clinical presentation.

It is recommended that patients presenting with an acute respiratory illness should be assessed and the following interrogated (Hess 2001:1276–1293):

- What is the rationale for ACT – is the pathophysiology potentially amenable to treatment?
- What is the potential for adverse effects of ACT?
- Which modality is likely to give the greatest benefit, with the least harm?
- What is the cost of ACT equipment and treatment?
- What does the patient prefer (even infants can display preference)?

If a decision is made to implement ACT, at the very least the effects of the therapy should be assessed and documented. Short-term outcomes may not be the most relevant, unless it can be shown that ACT makes the child more comfortable, or in the case of large segment lung collapse, where short-term effects of re-expansion could contribute significantly to overall outcome. More relevant would be to focus outcome measurement on disease progression, quality of life and patient or parental satisfaction levels (Hess 2001:1276–1293). If the patient is not improving, or is getting worse with ACT, the author suggests that physiotherapists should stop treatment or change treatment strategy. The vast majority of children with acute respiratory disease will recover completely without any intervention (De Boeck et al. 2008:607–612). Therefore, we should also consider Hippocrates’ wise statement – ‘to do nothing is also a good remedy’.

## Conclusions and recommendations

Airway clearance therapy should not be performed routinely in children admitted to hospital with acute respiratory conditions. All patients should be comprehensively assessed and treatment planned according to individual presentation, in those presenting with signs and symptoms that are potentially amenable to ACT.

There is an urgent need for rigorous clinical trials to develop evidence-based practice guidelines for ACT in acutely ill infants and children, and to examine the impact of ACT modalities on clinically relevant patient outcome measures.

In the words of Wallis and Prasad (1999), until that evidence becomes available, as physiotherapists:

*…* involved in the management of paediatric respiratory disorders [we] should avoid the unnecessary distress to both the child and family of useless treatment and the potentially serious consequences of inappropriate intervention. (pp. 393–397)
